# Supplementary material for: Human and Mouse Bone Marrow CD45+ Erythroid Cells Have a Constitutive Expression of Antibacterial Immune Response Signature Genes
Source: Biomedicines. 2025 May 17;13(5):1218. doi: 10.3390/biomedicines13051218 (PMC12108605; doi:10.3390/biomedicines13051218)
Supplement: Supplementary file 1 [file biomedicines-13-01218-s001.zip › Supplementary Figures S1-S2.pdf]

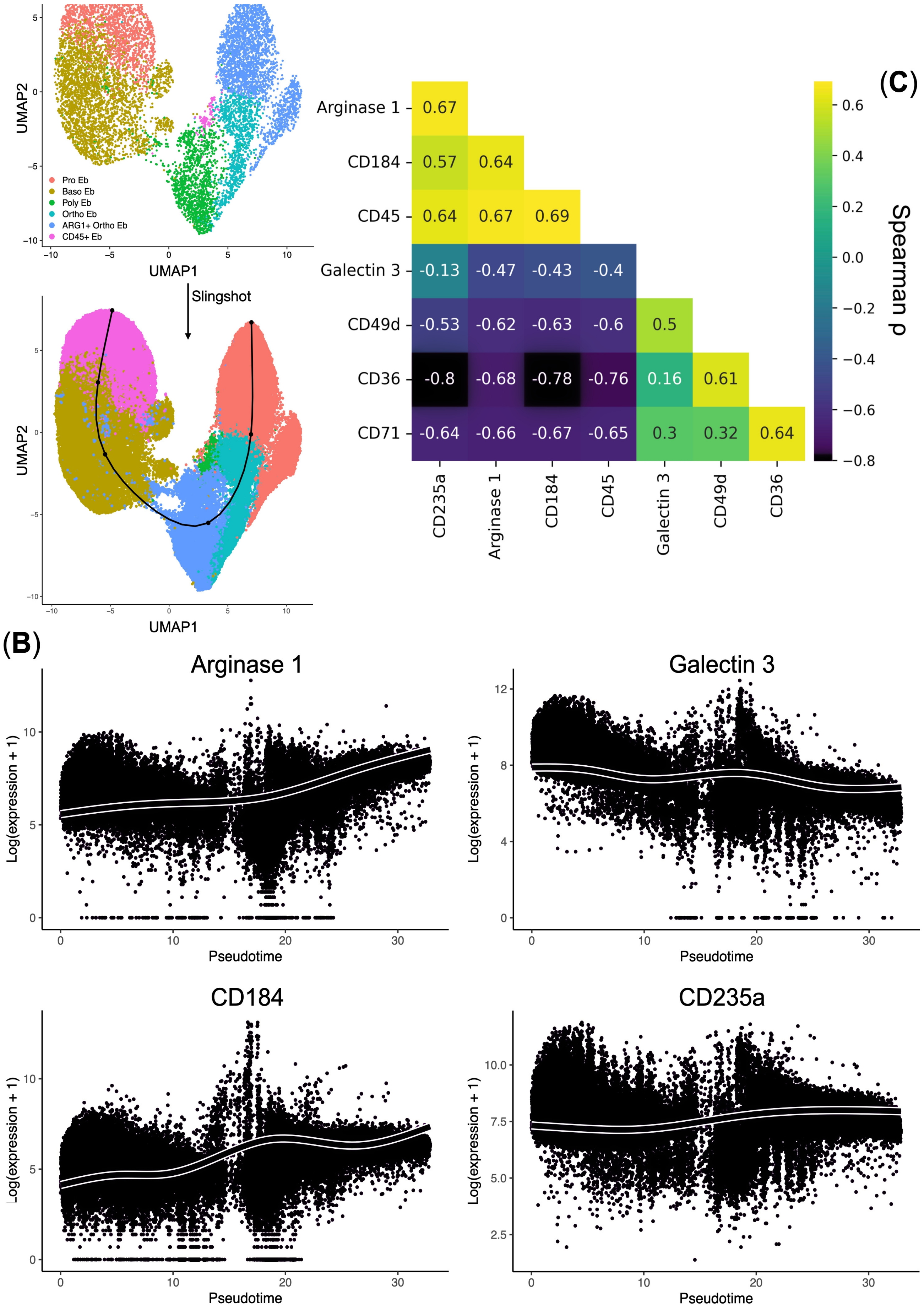

Figure S1: Pseudotime and Spearman correlation analyses of Erythroid-cell marker expression.

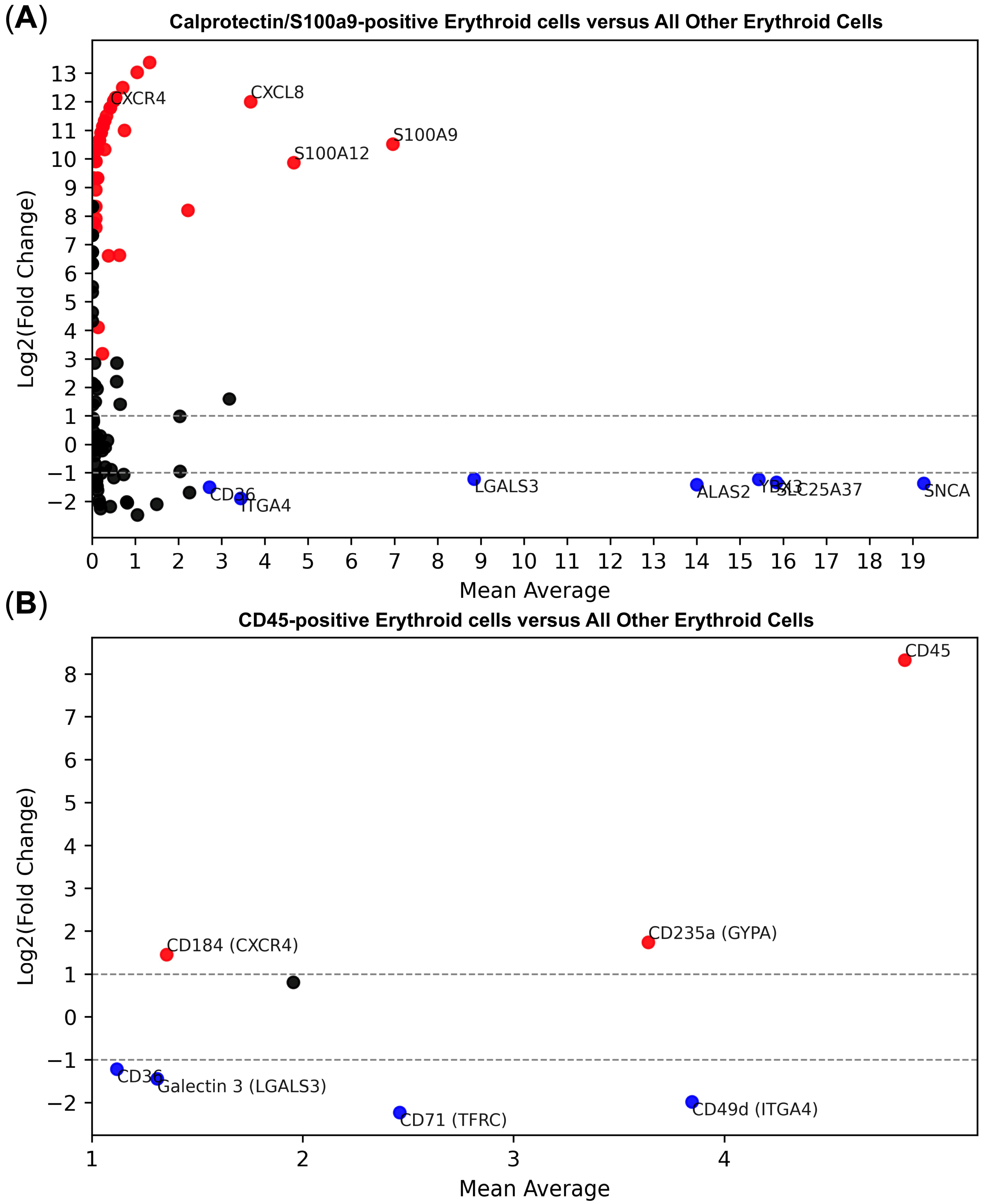

Figure S2: Differential expression of surface proteins and genes of CD45-positive Erythroid cells versus all other Erythroid cells.
